# Progress of Polyaniline Glucose Sensors for Diabetes Mellitus Management Utilizing Enzymatic and Non-Enzymatic Detection

**DOI:** 10.3390/bios12030137

**Published:** 2022-02-22

**Authors:** Velia Osuna, Alejandro Vega-Rios, Erasto Armando Zaragoza-Contreras, Iván Alziri Estrada-Moreno, Rocio B. Dominguez

**Affiliations:** 1 CONACYT-CIMAV, SC, Av. Miguel de Cervantes #120, Chihuahua C.P. 31136, Mexico; velia.osuna@cimav.edu.mx (V.O.); ivan.estrada@cimav.edu.mx (I.A.E.-M.); 2 Centro de Investigación en Materiales Avanzados, SC, Av. Miguel de Cervantes #120, Chihuahua C.P. 31136, Mexico; alejandro.vega@cimav.edu.mx (A.V.-R.); armando.zaragoza@cimav.edu.mx (E.A.Z.-C.)

**Keywords:** glucose monitoring, polyaniline, diabetes mellitus, enzymatic, non-enzymatic

## Abstract

Glucose measurement is a fundamental tool in the daily care of Diabetes Mellitus (DM) patients and healthcare professionals. While there is an established market for glucose sensors, the rising number of DM cases has promoted intensive research to provide accurate systems for glucose monitoring. Polyaniline (PAni) is a conductive polymer with a linear conjugated backbone with sequences of single C–C and double C=C bonds. This unique structure produces attractive features for the design of sensing systems such as conductivity, biocompatibility, environmental stability, tunable electrochemical properties, and antibacterial activity. PAni-based glucose sensors (PBGS) were actively developed in past years, using either enzymatic or non-enzymatic principles. In these devices, PAni played roles as a conductive material for electron transfer, biocompatible matrix for enzymatic immobilization, or sensitive layer for detection. In this review, we covered the development of PBGS from 2015 to the present, and it is not even exhaustive; it provides an overview of advances and achievements for enzymatic and non-enzymatic PBGB PBGS for self-monitoring and continuous blood glucose monitoring. Additionally, the limitations of PBGB PBGS to advance into robust and stable technology and the challenges associated with their implementation are presented and discussed.

## 1. Introduction

Diabetes Mellitus (DM) is a metabolic disorder characterized by the presence of hyperglycemia due to defects in insulin secretion, insulin action, or both [1]. The complications of DM include nephropathy, neuropathy, retinopathy, foot damage, skin conditions, increased risk of cardiovascular disease, and dementia, to name a few. Moreover, DM can be classified into four categories [2]:

1. Type 1 diabetes (T1DM): Related to autoimmune *β*-cell destruction, commonly leading to absolute insulin deficiency.
2. Type 2 diabetes (T2DM): Caused by a progressive loss of adequate *β*-cell insulin secretion, usually on the background of insulin resistance.
3. Gestational DM: High glucose levels diagnosed in the second or third trimester of pregnancy without having a prior diabetic history.
4. Other specific types of diabetes, e.g., monogenic diabetes syndromes, diseases of the exocrine pancreas, and drug- or chemical-induced diabetes.

Currently, 537 million working-age people (20–79 years) have diagnosed or undiagnosed DM. This number is projected to increase to 643 million and then to 783 million by 2030 and 2045, respectively [3]. In addition, the most significant increase will occur in regions where economies move from low to middle income status. The alarming increase in the worldwide prevalence of DM has increased interest in developing easy-to-use devices to support diagnosis and treatment. These devices require accurate self-assessment of glucose, the so-called golden standard for DM monitoring. Specifically, continuous glucose monitoring is a tool that helps significantly to prevent the risks of other diabetes-induced comorbidities, allowing people to maintain a healthy lifestyle, avoiding costly and lethal late-stage diabetic complications [4,5]. In 1962, Clark and Lyons manufactured the first enzymatic glucose sensor, which established the basis of self-assessment glucose monitoring. The fundamental principle is based on the control of oxygen consumption as a function of the catalytic oxidation of glucose by glucose oxidase (GO_x_) enzyme [6]. While the sensor presented several drawbacks, this breakthrough advance evolved into a global, successful, and well-established market. Since then, several innovative configurations and materials have been incorporated for glucose sensors including, metal nanoparticles [7], carbon-based nanomaterials [8,9], metallic oxide [10], and conductive polymers (CPs) [11,12,13].

**Figure 1:**
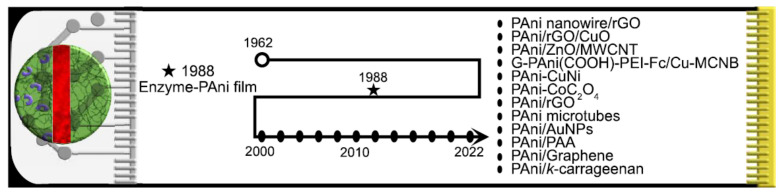
Graphical description of PAni-based glucose sensors (PBGS) evolution.

CPs such as polypyrrole (PPy), polythiophene, poly(3,4-ethylenedioxythiophene) (PEDOT) have been incorporated in novel glucose sensors; however, PAni has been extensively applied for chemical sensors and is closely related to the glucose sensing field. The application of PAni in chemical sensing arose in the mid-1980s when the first sensor for pH monitoring appeared [4]. At the end of this decade, the first studies of PAni-based glucose sensors (PBGS) also emerged, with Shinohara et al. reporting platinum electrodes and microelectrodes modified with electropolymerized PAni in the presence of GO_x_. In this case, the PAni allows enzyme stability and functions as a charge transfer interface between the enzyme and the electrode [5]. Figure 1 presents a graphical description of PBGS evolution, encouraged by CPs’ Nobel award, and formally started with Shinohara sensor device. Since then, there has been an increasing trend for the development of PBGS with novel morphologies and assemblies using PAni as base material. In this review, we covered the development of PBGS from 2015 to the present, using enzymatic and non-enzymatic mechanisms. We first introduced the principles of glucose mechanisms in both configurations, then we presented a background of the main PAni characteristics and their suitability for glucose detection. Subsequently, we collected references for enzymatic and non-enzymatic PBGS, highlighting the main achievements of these devices for glucose detection intended for DM care.

## 2. Glucose Sensing: Mechanisms for Enzymatic and Non-Enzymatic Detection

### 2.1. Mechanisms for Glucose Detection

**Figure 2:**
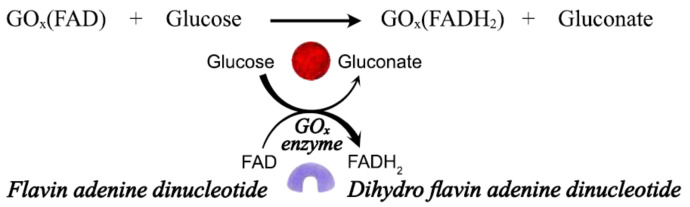
Mechanism for oxidation of glucose by glucose oxidase (GO_x_) enzyme.

The GO_x_ enzyme has been the most widely employed in enzymatic biosensors due to its relatively high selectivity, sensitivity, and stability. The main component of the large protein molecule is the redox center, flavin adenine dinucleotide (FAD). This redox center is located inside the enzyme, protected by a thick layer of protein. The FAD is reduced by interacting with glucose to dihydro flavin adenine dinucleotide (FADH_2_), producing the subproduct gluconate (Figure 2). The electron transfer to the active center is a major limiting factor and the cause of complicated electron transfer mechanisms. There are currently two approaches for oxidizing the reduced FADH_2_ center: Catalytic regeneration, reaction with a mediator, and direct electron transfer to the electrode (DET). Therefore, the glucose biosensor based on GO_x_ has evolved in three generations, and a fourth has come along without involving a biomolecule. In the following sections, each strategy will be discussed.

#### 2.1.1. First-Generation Biosensors

The first-generation of glucose biosensor is based on the catalytic regeneration of the FAD center in the presence of oxygen as a co-substrate. The first oxygen electrode was developed by Clark and Lyon [6], using GO_x_ coated by a semipermeable dialysis membrane on an electrode.

**Figure 3:**
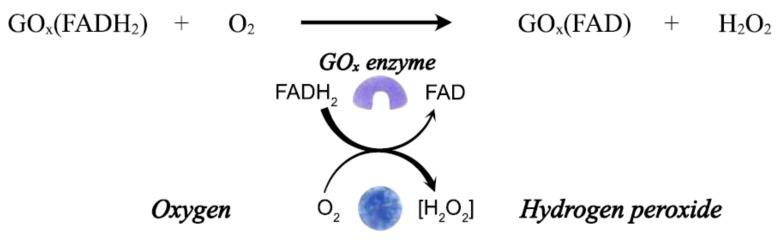
Reduction of GO_x_ (FADH_2_) by O_2_ for first-generation glucose biosensors.

The dependence on free oxygen as a catalytic mediator and electroactive interference species in the blood are two main problems this biosensor faces. For instance, there is a low concentration of oxygen in blood samples, which is not enough to maintain glucose oxidation efficiently; so, this oxygen deficit has a significant impact on the accurate determination of glucose levels. The associated mechanism for first-generation enzymatic biosensors is shown in Figure 3.

#### 2.1.2. Second-Generation Biosensors

Due to the oxygen dependence problems and electroactive interferences observed in first-generation enzymatic glucose sensors, alternative co-substrates were used, namely mediators. Furthermore, the synthetic electron acceptance mediators make it easier to transfer electrons from the enzyme’s redox-active site to the conductive electrode and subsequent reoxidation by the electrode, resulting in a quantifiable current. Several non-physiological mediators have been studied, including those derived from ferrocene [14] and ferricyanide [15], quinones [16], and transition metal complexes [17].

Moreover, the mediators generally have insolubility and low molecular weight characteristics, making them ideal for enzymatic glucose analysis. In addition, mediators possess reversible or quasi-reversible properties, a lower redox potential to avoid the oxidation of the interfering species, high stability, resistance to the formation of secondary compounds, and low toxicity. Nevertheless, it is complicated to maintain contact between species (enzyme-mediator-electrode) because the mediators are small and more diffuse during relatively prolonged device use [15,16].

Another drawback is that the mediator reacts with the enzyme at a rate considerably faster than oxygen. However, it causes a competitive reaction between the dissolved oxygen-enzyme and the mediator-enzyme, thus reducing the efficiency of the system and causing an accumulation of hydrogen peroxide.

Over the years, a variety of organic and inorganic compounds have been studied as electron transfer mediators. For instance, osmium complexes [18,19], ferricyanide [20,21], and their derivatives meet the mainly required criteria for an excellent mediator. These requirements are low operational potential, high chemical content, stability of both redox forms, rapid reaction with reduced enzyme (to minimize O_2_ competition), and low solubility in aqueous media.

In recent years, the enzymatic glucose sensor developed by Heller et al. [22] for Abbott Diabetes Care is a clear example of this second generation [23]. The FreeStyle blood is a typical example of the second-biosensors generations because it employs the glucose dehydrogenase (GDH) enzyme instead of GO_x_, and osmium complex as mediator. In addition, the measurement method of the glucose monitoring system is microcoulometric and requires a small blood volume (0.3–4.0 μL).

#### 2.1.3. Third-Generation Biosensors

DET is the principle employed in the third generation of enzymatic glucose sensors. The DET from glucose to electrode surfaces through the enzyme’s redox center is a challenge that has long been pursued. However, difficulties arise from the thick layer of insulating protein that surrounds the enzyme’s active center that hinder the efficient transfer of electrons with the surface of the electrode. Specifically, for GO_x_, the FAD/FADH_2_ center is located at a depth of about 13 Å within the apoenzyme. The advantages of the DET mechanism include removing artificial leachable mediators and operating in a potential window close to the redox potential of the enzyme.

Moreover, glucose and molecules with DET characteristics access this center through the diffusion and penetration of the 3D molecular network. Since this is not possible in a flat electrode, the inclusion of nano and porous materials has raised interest in increasing the surface area and electrode performance [24]. For example, electrodes with mesoporous materials exhibited a surface that traps and encompasses the enzyme; so, a DET from the enzyme to the electrode can occur, and a current related to the oxidation of the enzyme can be registered, without mediators, concerning the overlapping of electroactive interferences, or relying on dissolved oxygen. Nevertheless, the adverse effects of dissolved oxygen competing with the electrode to regenerate the enzyme have not yet been resolved. For this reason, the development of DET-based biosensors continues in the research stage and commercial glucose biosensors employ mainly first and second-generation electrochemical sensors [25].

#### 2.1.4. Non-Enzymatic Glucose Sensors (Fourth Generation)

The interest in non-enzymatic glucose sensors has grown due to the drawbacks of enzyme-based biosensors, such as the possible decrease in the catalytic activity of the enzyme derived from the immobilization process, limited reproducibility, and low stability during prolonged operation. The direct electrocatalytic oxidation of glucose occurs on the electrode surface to avoid deficiencies that originate in the nature of enzymes. A wide variety of materials have been utilized in non-enzymatic glucose sensors, e.g., CPs [11,12], multiwalled carbon nanotubes (MWCNT) [9,13], and molecularly imprinted polymers (MIPs) [26,27], which can mimic enzymes by creating crosslinked polymeric active sites for specific analytes.

The mechanism of non-enzymatic glucose oxidation on the electrode surface is not fully understood and remains an active area of research. However, two main models explain the principles: The first one was proposed by Pletcher [28] and is also known as the activated chemisorption model. In this, the oxidation is started by the adsorption of glucose on the electrode surface. In aqueous solutions, the two most common isomers are *α*-D-glucose and *β*-D-glucose, both in their cyclic hemiacetal glucose form. Immediately, the glucose molecule forms a physics interaction with the electrode surface, leading to glucose–metal interaction. Simultaneously, the hydrogen atom located in the hemiacetal carbon is removed from the glucose molecule and attaches to the electrode surface at a site adjacent to the glucose; this step determines the rate in most electrooxidation processes. Hence, the complex glucose–metal suffered desorption of the glucose molecule because of a change in the oxidation state of the glucose molecule.

**Figure 4:**
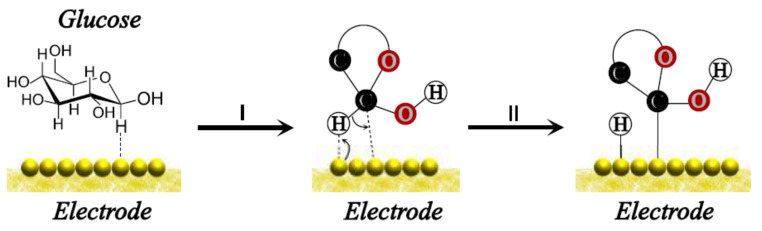
Illustration of the glucose–electrode interaction in the Pletcher model.

Therefore, the formation of intermediate strength is ideal for the adsorption and desorption process to occur. In fact, the proposal of active transition metal centers through the electrode does not consider the oxidative role of hydroxyl radicals that appear absorbed during the electrooxidation of glucose and other organic molecules [29]. A schematic proposal of this model is shown in Figure 4.

**Figure 5:**
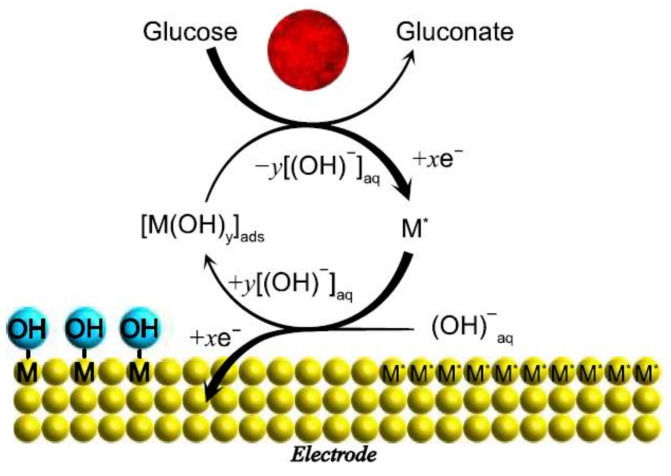
Illustration of the glucose-electrode according to incipient hydrous adatom mediator (IHOAM) model.

The second model is known as the “Incipient Hydrous Adatom Mediator” (IHOAM) model, Figure 5, and it is based on the active metal atoms at the electrode surface, which have low reticular stabilization and enhance reactivity. These metal atoms present an oxidation stage prior to the monolayer, during which an incipient hydrous oxide premonolayer (OH_ads_) is formed, which is suggested to mediate the oxidation of glucose on the surface of the electrode. While models can be used to explain glucose oxidation for different electrode materials, both models are still under study [30].

## 3. Polyaniline for Glucose Sensors

### 3.1. PAni Structure

**Figure 6:**
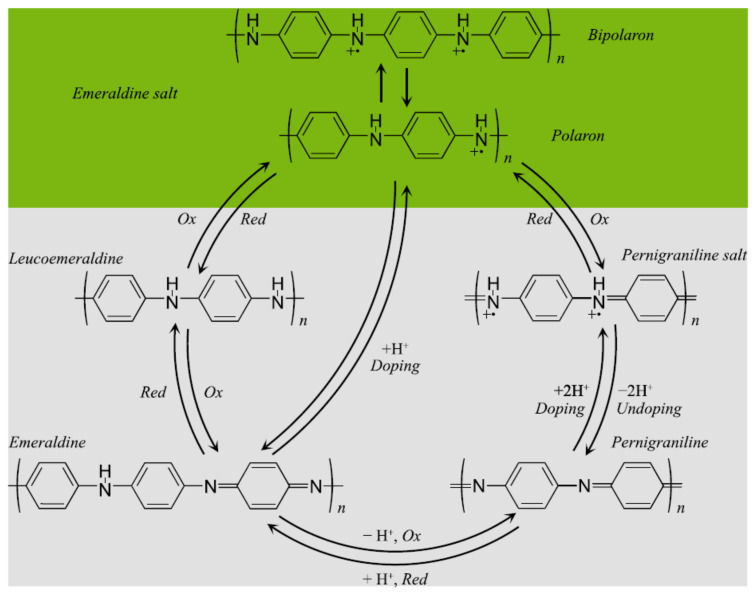
Basic polyaniline structures: Leucoemeraldine, Emeraldine salt, and Pernigraniline.

The application of CPs in the design of biosensors has been reviewed in detail on several occasions in the past [31,32]. Thanks to the ability to transfer electrons produced by biochemical processes, CPs offer significant advantages since they are sensitive to small amounts of the analyte [33]. PAni is a semi-flexible CP of most significant interest for biosensor design due to its electrochemical properties, e.g., reversible redox behavior and electrochemical tuning, simple processability, and long-term stability. Leucoemeraldine, emeraldine salt, and pernigraniline are the three fundamental oxidation states of PAni. Furthermore, it is a versatile material that can have diverse applications in essential areas of science and technology, including electrochromic devices, solar cells, actuators, tissue engineering, and biosensors. The basic PAni structures are shown in Figure 6, along with their doped and undoped forms.

### 3.2. Conductivity

In CPs, the oxidation state is decisive in their electrical properties; however, PAni has characteristics that differentiate it from the rest. In PAni, electrical conductivity depends on both oxidation state and doping. When PAni in the form of emeraldine base is exposed to a protonic acid (proton doping), nitrogen doping of the imine groups occurs, from which polaron delocalization occurs along the chain, obtaining the conductive form of PAni (emeraldine salt) [34,35]. Proton doping of PAni does not involve the loss or gain of electrons, which occurs in a redox process [36]. Such doping can cause an increase of up to 10 orders of magnitude in the electrical conductivity of PAni [37]. In CPs, the role of the electrons-hole pair is a determining factor in electrical conduction [38,39]. Compared with conventional metals, the CPs do not have a range of energies that are considered unavailable for electrons because the valence and conduction bands overlap. Hence, the band gap or energy gap (*E_g_*) in metals is 0 eV. Regarding conductive PAni, the energy gap is ~3.0 eV [40]. Under these conditions, electrons can increase their energy and detach themselves from their atoms by jumping to a higher energy level in the conduction band. For emeraldine base (non-conductive), the *E_g_* between the valence and conduction bands is too large, greater than 3 eV; consequently, the electron is not capable of making such a jump that allows it to ascend to the conduction band, showing poor electrical conduction. It should be noted that experimental and theoretical studies, despite some differences [41], propose that the electronic transport of emeraldine salt is based on polaronic and bipolaronic structures; however, even today, there is no complete consensus [42]. Through doping with a Brønsted acid, such as hydrochloric acid (HCl), the imine groups are protonated to form the bipolaron. The bipolaron then dissociates to form two polarons, which subsequently rearrange, forming a delocalized polaron in the form of a polysemiquinone (radical-cation salt) [43,44].

### 3.3. PAni as Matrix Enzyme Immobilization

The factors that influence the enzyme stability are polymerization conditions, incorporation of the enzyme into the matrix of the CP, and dispersion or solution of the enzyme. In particular, concerning PAni-based enzymatic biosensors, it is necessary to consider the polymerization conditions of PAni because emeraldine salt is obtained at pHs lower than 3 (highly conductive). Under these conditions, and in the presence of the polymerization by-products (traces of aniline salt, ammonium persulfate (APS)), enzymes can lose their catalytic activity. On the other hand, the method of incorporation of the enzyme into the CP can also affect the enzyme stability and, therefore, the operation of the sensor. For example, Mu and Xue [45] compared the performance of GO_x_ in electrochemical glucose sensors, as it was supported on the PAni by physical absorption or by in situ polymerization in the presence of the enzyme. The remarkably superior performance of the sensor with the electrochemically deposited enzyme was attributed to the electrochemical doping because the enzyme is strongly bound to the CP through ionic bonds and the desorption towards the electrolyte is lower than when the enzyme is only physically deposited [45]. Other strategies have also been applied to maintain enzyme activity. In this sense, Yan et al. reported an electrode modified with an Au nanoparticles (NPs)–AgCl–PAni biocompatible substrate, on which GO_x_ was immobilized. Electrochemical studies using pH 5 to 9 PBS showed excellent enzymatic activity at pH 6, which is generally adverse for PAni [46]. Subsequently, Zhao et al. reported the modification of an electrode with PAni nanofibers (NFs) synthesized via oxidative polymerization in an interfacial system. GO_x_ was covalently bonded to the NFs through amidation reactions. Circular dichroic and cyclic voltammetry (CV) measurements showed that the covalent immobilization did not affect enzymatic activity [47]. Finally, the use of mixtures of organic solvents with water has also been a tactic used to achieve enzyme stability, e.g., GO_x_, in the sensor [48], as reported by Lukachova et al., who used water–ethanol mixtures with high ethanol contents. The enzymatic stability in these mixtures and in the presence of a Nafion emulsion as a solid electrolyte was higher than in pure water because the enzyme loses its solubility while remaining in suspension. By this procedure, the enzymatic stability of GO_x_ can be improved compared to traditional methods using highly dilute electrolytes [49]. The subject of enzymatic glucose sensors is discussed more fully later.

### 3.4. Biocompatibility for Implantable Devices

The requirements of continuous in vivo glucose monitoring in patients with critical conditions of DM have stimulated the development of implantable sensors. Since its inception, the biocompatibility or tolerance of body fluids or tissues with the materials that make up the biosensor has been an aspect of the in-depth study [50]. In addition to interfacial biocompatibility, it is also desirable that body fluids do not cause surface obstruction of the sensor to develop continuous sensing for long periods [51]. It is vital that the patient is not exposed to any danger due to some reaction derived from the sensor implantation, e.g., blood clotting or inflammatory response, because some sensor surface characteristics such as surface roughness and chemical factors such as wettability and surface charge can stimulate poor hemocompatibility that can cause such adverse conditions [52]. CPs also have excellent biocompatibility, as demonstrated by Sun et al. for PAni hydrogels, whose harmlessness towards rat endothelial progenitor cells was demonstrated. In addition to non-toxicity, the PAni hydrogel showed improved cell adhesion and a faster cell proliferation rate [53]. Similarly, Humpolicek et al. [54] conducted biocompatibility studies of PAni, in its conductive and non-conductive forms, with human cell lines. Studies indicated that none of the forms of PAni caused skin damage; however, the emeraldine salt form was found to exhibit high cytotoxicity. The subsequent purification of the materials showed a considerable reduction in cellular incompatibility, which indicated that the by-products of polymerization are responsible for this effect [54]. Another study conducted in more detail by Kašpárková et al. confirmed that residues of APS, aniline, or aniline salt are highly cytotoxic; therefore, the purification of PAni is of utmost importance towards biomedical applications [55].

## 4. Polyaniline Based-Enzymatic Glucose Devices

### 4.1. PAni-Based Glucose Biosensors

**Figure 7:**
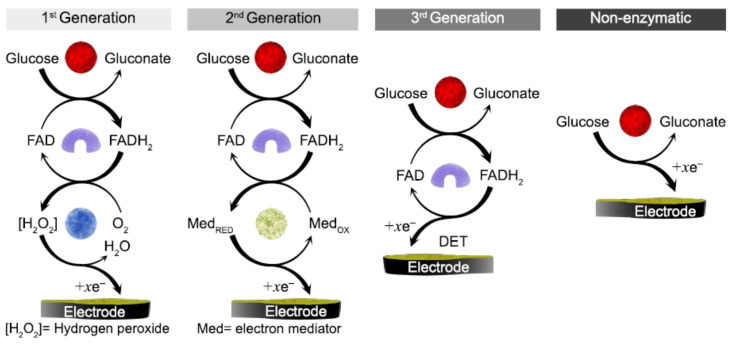
Electrochemical PBGS: First generation, second generation, third generation and non-enzymatic.

Accurate glucose monitoring is a crucial aspect of proper DM management, with enzymatic sensors playing a prominent role in this task. Enzymatic glucose sensors rely on a redox reaction performed on the surface of the electrode driven by the GO_x_ enzyme. The commonly found electrode materials include glassy carbon electrodes (GCE) [56,57] screen-printed carbon electrodes (SPCE) [58,59] and Platinum (Pt) electrodes [60,61]. In PBGS, these supports are subsequently modified with PAni obtained by a variety of methodologies such as chemical oxidative polymerization (COP) [56], electrochemical polymerization (ECP) [62], free radical polymerization [59], plasma polymerization [63] and interfacial polymerization [64]. The sensing mechanisms of PBGS have followed the innovation path described in Section 2.1, acting as a transducer for detecting either O_2_ or H_2_O_2_ subproducts in the first generation, as electron mediator in the second generation, and even as a molecular wire for DET in the third generation. Figure 7 shows the mechanism of detection followed by electrochemical PBGS in all generations.

**Figure 8:**
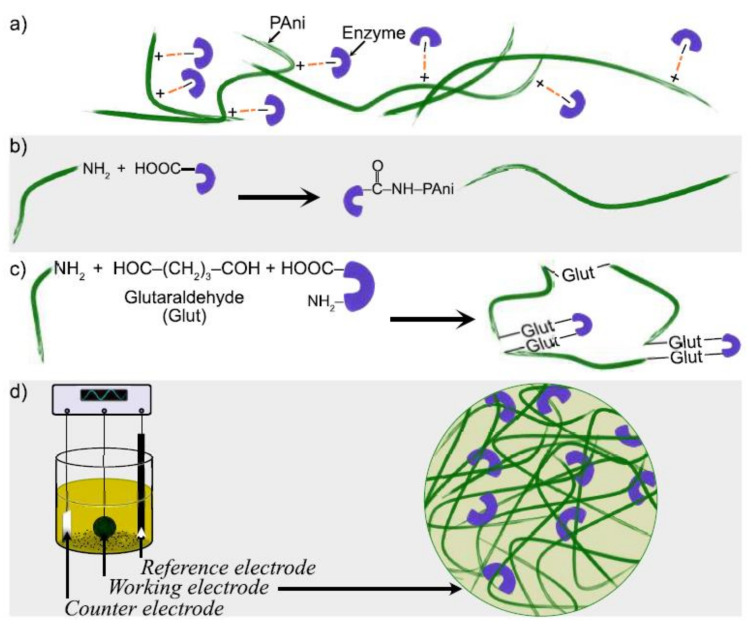
Immobilization strategies for GO_x_ in PAni-based biosensors: (**a**) Physical absorption, (**b**) covalent bonding, (**c**) crosslinking, (**d**) entrapment by ECP.

A key aspect for optimal performance of electrochemical biosensors is the proper immobilization of enzymes over the electrode, which can be achieved through physical and chemical strategies [65]. When PAni is incorporated over the working electrode, the modified surface exhibits suitable characteristics for enzyme immobilization such as enhanced conductivity, high surface area, available functional groups for linkage and biocompatibility. The most straightforward immobilization technique for PBGS is physical absorption of the GO_x_ through electrostatic attraction and Vander Walls forces; however, the poor long-term operational stability is a major drawback of this technique [66]. The immobilization by crosslinking with glutaraldehyde or covalent bonding through the reaction with amino (NH_2_) functional groups allows high enzyme loading and enhances operational stability. Nevertheless, the chemical reactions can lead to structural changes of the enzyme [67]. A convenient and highly reported immobilization method is the direct entrapment of the enzyme during the ECP of PAni. In this one-step preparation method, the aniline monomer and GO_x_ are in the same solution, producing biofilms on the electrode surface by controlling applied potential, time, and enzyme amount [65]. A schematic representation of the main steps of the immobilization strategies for GO_x_ using PAni is presented in Figure 8.

In addition to the immobilization matrix, a prominent role of PAni is as electrode material for electrochemical transduction. Enzymatic glucose biosensors with bare PAni as transducer take advantage of the polymer key features such as high conductivity, inherent electroactivity and adequate support for electronic transfer. Electrodes modified with sole PAni can exhibit different morphologies such as microtubes [68], nanofibers, nanoflakes [69], and nanoflowers [70], acting as transduction elements and immobilization matrix for GO_x_ at the same time. However, the results for PAni as a single electrode element still present challenges, including limited detection range and low operational stability in the long term. To improve these issues, copolymerization has been proposed for tuning specific characteristics of PAni, e.g., conductivity [64] or solubility [71] of the polymer. For example, to improve the processability of PAni, Tugrul Cem Biak developed a PAni-poly(ethylene glycol) graft copolymer (PAni-g-PEG) combining COP and click chemistry. The resulting water-soluble copolymer allowed a simpler method for GO_x_ immobilization through electrostatic interaction and hydrogen bonding, simplifying the biosensor manufacturing process [71].

**Figure 9:**
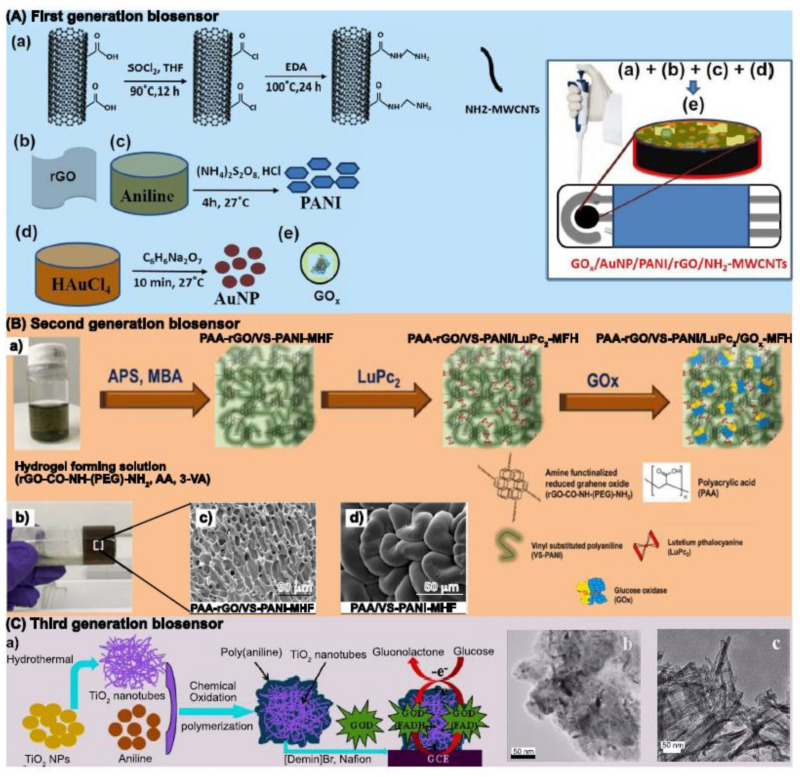
Applications of PAni in enzymatic biosensors: (**A**) Preparation of NH_2_-MWCNT/PAni/rGO/AuNPs/GOx first generation biosensor: (**a**) synthesis of NH_2_-MWCNT, (**b**) rGO, (**c**) COP of PAni, (**d**) synthesis of AuNPs, (**e**) assembly of NH_2_-MWCNT/Pani/rGO/AuNPs for GOx immobilization; (**B**) the assembly of PAA/rGO/VS-PAni/LuPc_2_/GOx/MHF for second generation biosensor (**a**), a view of the obtained MFH (**b**) along with SEM images of the morphology of PAA/rGO/VS-Pani/MHF (**c**) and MHF morphology without rGO (**d**); (**C**) synthesis of PAni/TiO_2_NT composite for third generation biosensor (**a**), and SEM images of PAni (**b**) and TiO_2_NT (**c**). Figures reproduced with permission from Debasis Maity et al., H. Al-Sagur et al., and Jie Zhu et al. [58,59,81].

However, for developing PBGS, the most widely reported strategy is the assembly of ternary and quaternary composites with a plethora of materials, including carbon-based nanomaterials (e.g., graphene [72,73], MWCNT [57,74]), metallic nanoparticles (e.g., Au [57,75], Pt [76,77]), and polymers (e.g., PEO [78], polyacrylic acid [79], and SDS [80]). An example of these strategies was presented by Maity et al., developing a composite of aminated (NH_2_) MWCNT/PAni/rGO/AuNPs over an SPCE [58]. PAni was synthesized by COP and mixed by sonication with NH_2_-MWCNT, rGO, and AuNPs. The obtained nanocomposite exhibited convenient characteristics such as high surface area, porosity, and available sites for GO_x_ covalent immobilization (Figure 9A). In this case, PAni acted as a conductive binder of the assembly over the surface of SPCE. The NH_2_-MWCNT/PAni/rGO/AuNPs/GO_x_ biosensor showed good electrocatalytic activity, following the detection mechanism described by first glucose biosensor generation. The obtained linear detection range was 1 to 10 mM, a sensitivity of 246 μA mM^−1^ cm^−2^, and a limit of detection (LOD) of 63 μM. Additionally, the biosensor performed well in the presence of interferences such as dopamine, uric acid, and ascorbic acid. The device was evaluated in blood serum samples for practical applications, achieving a recovery rate above 96.5% and retaining 93.4% of the initial activity after 60 days, when stored at −20 °C.

In a different strategy, H. Al-Sagur et al. reported a three-dimensional hydrogel assembled with polyacrylic acid (PAA), aminated RGO, vinyl substituted PAni (VS-PAni), and lutetium Phthalocyanine (LuPc_2_), Figure 9B [59]. The so-called multifunctional hydrogel (MFH) was obtained using free radical polymerization by incorporating VS-PAni with rGO and PAA during the synthesis. Then, the MFH was cast over an SPCE and subsequently doped with LuPc_2_ to be applied as support for GO_x_. The characterization revealed a hierarchical structure with low charge transfer resistance and interconnected networks of VS-PAni. The proposed mechanism corresponds to the second generation, with LuPc_2_ acting as a transfer mediator. The PAA/rGO/VS-PAni/LuPc_2_/GO_x_/MHF biosensor resulted in a linear range for glucose detection from 2 to 12 mM, a sensitivity of 15.31 μA mM^−1^ cm^−2^, and LOD equal to 25 μM. The device exhibited a fast response of around 1 s, remarkable stability for three months when stored at 4 °C and was successfully evaluated in clinical serum samples. Additionally, PAni-based composites have been applied for DET in third-generation biosensors. Jie Zhu et al. developed a PAni-TiO_2_NT composite, departing from TiO_2_NT synthesized by hydrothermal method and subsequently polymerizing PAni by COP (Figure 9C) [81]. A suspension of the PAni-TiO_2_NT composite, GO_x_ and Nafion was mixed and cast over a GCE to create the glucose biosensor. The electrochemical characterization revealed the DET of immobilized GO_x_ produced by electron exchange between FAD and the PAni-TiO_2_NT/GCE electrode. Overall, the biosensor exhibited a narrower linear range than previous generations, from 10 to 2500 μM, a sensitivity of 11.4 μA μM^−1^, and a low LOD of 0.5 μM. The current decreased less than 10% after one month, which was attributed to the excellent biocompatibility of the composite. Furthermore, the described representative cases, the Table 1 presents a more comprehensive revision from 2015 to date of enzymatic PBGS.

### 4.2. PAni-Based Implantable Glucose Biosensors

**Figure 10:**
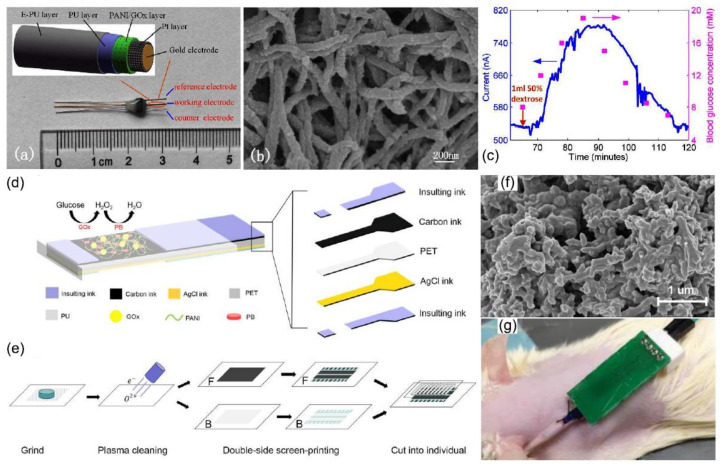
Implantable PAni-based biosensors for continuous glucose monitoring: (**a**) A view of the CAD design of the Pt/PAni/GOx/PU/E-PU biosensor and the real implantable device, (**b**) SEM view of the PAni NF immobilization matrix, (**c**) recorded subcutaneous glucose response (blue) for in vivo test against blood glucose measurement, (**d**,**e**) fabrication of PB doped/PAni/GOx/PU biosensor, (**f**) SEM view of GOx immobilized in PAni matrix, (**g**) implanted device in rat specimen for in vivo test. Images reproduced from references Lu Fang et al. [82] and Yu Cai et al. [83].

So far, taking glucose measurements at a given time has been the classical approach for monitoring; however, the needs of diabetics healthcare require a focus on a continuous approach. The materials associated with continuous and implantable devices need to exhibit superior biocompatibility as well as avoid the biofouling of the device. In this sense, PAni has been evaluated as a material for in vivo detection of subcutaneous glucose, Table 1. A biosensor presented by Lu Fang et al. was developed with an arrangement of several layers: Electrodeposited Pt layer, a PAni/GO_x_ layer, Polyurethane (PU) layer, and Epoxy-PU layer (E-PU) [82], Figure 10. In particular, PAni was electropolymerized over the working electrode, and GO_x_ was immobilized by crosslinking. PU and E-PU provided stability and biocompatibility as well as a protective layer for the implantable sensor. First, the Pt/PAni- GO_x_/PU/E-PU biosensor was evaluated in PBS and bovine serum, showing similar analytical parameters of sensitivity (63 nA/mM) and working range (0–20 mM). Then, the device was tested for in vivo implantation in rats connecting the electrodes under the skin and recording the signals produced by dextrose injection. The recorded in vivo values showed good accordance with blood glucose obtained from the vein tail, observing a delay of 8 min attributed mainly to physiological phenomena in the implanted tissue. The biosensor showed an in vivo lifetime of 26 days, with a fluctuation period during the first 12 days, followed by a stable period from day 12 to 26. The authors attributed the extensive life to excessive GO_x_ immobilized in the PAni NF matrix and the protective PU/E-PU membrane. In a different approach, a flexible, double-sided SPCE was used as the base of a PB doped/PAni/GOx/PU biosensor [83]. In this design, the front was manufactured with a carbon ink doped with PB as the working electrode, and the back was printed with Ag/AgCl ink as the counter electrode. Then, the carbon/PB electrode was modified with PAni and crosslinked GO_x_, and finally covered with a protective PU layer. The cytotoxicity study revealed the benefits of PU coating for the working electrode composite even though it showed minimal effect for the highly toxic Ag/AgCl electrode. The PB doped/PAni/GO_x_/PU also showed advances in terms of manufacturing; nevertheless, the in vivo experiments showed that the information recorded by the implantable sensor needs improvements to correlate with registered blood glucose.

**Table 1:** Enzymatic PAni-based glucose sensors (PBGS).

Gen	Material	Immob. Method	Polymerization	Technique	Linear Range	Sensitivity μA·mM^−1^·cm^−2^	LOD μM	Sample	Stability	Ref.
3rd	TiO_2_/PAni	entrapment in chitosan	vapor phase polymerization	AMP	20–140 μM	163.14	5.33	-	-	[84]
	PAni-CoC_2_O_4_	covalent bonding	COP	AMP/ DPV	3–13 mM	0.099 *	94	-	-	[85]
	PAni/MWCNT/AuNPs	entrapment in chitosan	-	AMP	0.063–1.19 mM	29.17	0.21	serum	-	[57]
	PAni/activated carbon/TiO_2_	crosslinking	COP	AMP	0.02–6.0 mM	-	18	-	-	[86]
	PAni microtubes	electrostatic attraction	in situ polymerization	AMP	4–800 μM	35.42	0.8	serum	-	[68]
	PAni/TiO_2_ NT	entrapment	COP	DPV	10–2500 μM	11.4 *	0.5	-	2 months	[81]
	PAni-PVP-AuNPs/GOx/Nafion	physical absorption	ECP	AMP	0.05–2.25 mM	9.62	10	serum	2 weeks	[75]
	PAniNW/rGO	electrostatic interaction	galvanostatic polymerization	AMP	0.1–8.5 mM	12.3	0.1	-	25 days	[87]
2nd	PAni/rGO	crosslinking	interfacial polymerization	AMP	0.5–50 mM	2.8*	89	serum	8 days	[73]
	PAni	EC entrapment	ECP	potentio metric	0.1–100 mM	14.6 mV/ decade	-	-	-	[88]
	PAni	EC entrapment	ECP	EIS	0.1–100 mM	7.4%/ decade	0.98	-	-	[89]
	PAA-VS-PAni/GPL-FePc	crosslinking	COP	AMP	1–20 mM	18.11	6.4	serum	-	[90]
	SiO_2_-LuPc_2_/PAni-PVIA-CNB	crosslinking	COP	AMP	1–16 mM	38.53	1000	serum	45 days	[91]
	PAA-rGO/VSPAni/LuPc_2_	crosslinking	free-radical polymerization	AMP	1–12 mM	15.31	25	serum	3 months	[59]
	PAni/PEO	physical absorption	rapid mixing polymerization	AMP	1–10 mM	16.04	820	-	15 days	[78]
	PAni/poly(acrylic acid)	physical absorption	ECP	AMP	up to 40 mM	-	-	-	-	[79]
1st	PAni/AuNPs	crosslinking	COP	AMP	up to 16.5 mM	65.4	70	serum	22 days	[92]
	PAniHF/PtNPs	crosslinking	in situ polymerization	AMP	0–24 mM	35 **	-	blood	7 days	[77]
	PAni	potentiostatic entrapment	ECP	AMP	0.01–0.1 M	-	-	-	-	[93]
	NH_2_-MWCNT/rGO/PAni/ AuNPs	physical absorption	COP	AMP	1–10 mM	246	64	Blood serum	30 days	[58]
	PAni-CNT	covalent bonding	ECP	DPV	2–426 μM	620	1.1	Blood plasma	45 days	[94]
	PAni-Montmonirollite-PtNPs	entrapment in chitosan	ECP	AMP	0.01–1.94 mM	35.56	0.1	blood serum	40 days	[60]
	PB doped ink/PAni/GOx/PU	physical absorption	COP	AMP	0–12 mM	16.66	-	*in vivo*	14 days	[83]
	Graphene/PAni-co-PDPA	physical absorption	interfacial polymerization	AMP	1–10 μM	0.51 *	0.1	serum	20 days	[64]
	PAN/PAni/Graphene	entrapment in chitosan	COP	AMP	0.01–1.97 mM	29.11	2.10	penicillium	1 month	[61]
	PAni/SnO_2_NF	entrapment in chitosan	COP	AMP	5–100 μM	-	1.8	urine	60 days	[56]
	PAni-SDS-F127	crosslinking	in situ polymerization	AMP	5–50 mM	485.787	3.202	-	-	[80]
	PAni/PAA	covalent bonding	ECP	AMP	0–16 mM	49.3	26.5	-	-	[95]
	PAni/SnO_2_@3D-rGO	physical absorption	plasma polymerization	AMP	0.56 nM–270 μM	-	0.26 nM	serum	1 month	[63]
	PAni-g-PEG	crosslinking	COP/Click chemistry	AMP	0.05−1.0 mM	47.72	20	-	20 days	[71]
	PaniNFW/nanodiamond	crosslinking	graft polymerization	AMP	1–30 mM	2.03 *	18	serum	30 days	[70]
	PAniNFs/GOx/PU/E-PU	crosslinking	ECP	AMP	0–20 mM	63 **	-	*in vivo*	14 days	[82]
	MWCNT-COOH/PAni	covalent bonding	COP	FET	0.005–500 mM	-	0.5	serum	1 week	[96]
	MWCNT/Au NPs/PAni	physical absorption	in situ polymerization	CV	2–12 mM	12.73	-	-	2 days	[97]
	4-amino thiophenol/Au NPs/GOx–HRP/6-mercapto-1-hexanol-11-mercaptoundecanoic acid/Au	covalent bonding	enzymatic polymerization	AMP	0.0165–10.0 mM	41.78	5.4	serum	10 days	[98]
	Graphene/PAni	entrapment in chitosan	ECP	AMP	0.01–1.48 mM	22.1	2.769	plasma	-	[72]
	3D PB/PAni/MWCNT	crosslinking	COP	AMP	0.05–4 mM	-	40	in vivo	1 h	[99]
	AuNPs/PAni-Pt NPs/GOx/PVDF-Nafion	EC entrapment	ECP	AMP	0–20 mM	0.23 *	-	in vivo	21 days	[76]
	PB/IL-PAni/MWCNT	entrapment in chitosan	ECP	AMP	0.0125–1.75 mM	94.79	1.1	serum	-	[62]
	PAni/k-carrageenan	physical absorption	COP	DPV	1–15 mM	0.86	-	-	-	[100]

COP: Chemical oxidative polymerization; ECP: Electrochemical polymerization; EC entrapment: Electrochemical entrapment; AMP: Amperometry; CV: Cyclic voltammetry; DPV: Differential pulse voltammetry; * μA/mM; ** nA/mM. PAA-VS-PAni/GPL-FePc:polyacrilic acid (PAA)-vinyl substituted polyaniline (VS-PAni)/iron phthalocyanine functionalised graphene nanoplatelets (GPL-FePc). SiO_2_-LuPc_2_/PAni-PVIA-CNB:lutetium phthalocyanine incorporated silica nanoparticles (SiO_2_-LuPc_2_) grafted with (poly(vinyl alcohol-vinyl acetate) itaconic acid) doped polyaniline conducting nanobeads (PAni-PVIA-CNB).

## 5. Polyaniline-Based Non-Enzymatic Glucose Sensor

The design of novel nanomaterials for non-enzymatic glucose detection has raised considerable attention in recent years. The primary motivation is to avoid the drawbacks associated with enzymatic glucose operation, e.g., limited temperature conditions, narrow pH range, the need for a biocompatible environment, and operational instability after long-term storage. However, the proposed materials aiming to replace the action of GO_x_ must ensure a mechanism to replace the high selectivity of the enzyme; such sensors also need to function at physiological pH conditions and provide a proper cost-to-benefit operation. Moreover, as presented in Section 2.1.4, the non-enzymatic glucose sensing mechanisms are mainly described in alkaline conditions. So far, Pt and AuNPs are among the main reported materials for non-enzymatic detection, although transition metals, metal alloys, and carbon-based nanomaterials present promising advances [101]. Similarly, PAni-based for non-enzymatic glucose sensing has been increasingly reported during the last years. For example, PAni as electrocatalytic material for glucose oxidation was evaluated by Ameen et al. with nanocage-augmented PANI nanowires (NCaPAni NWs) [102]. During CV, the sensor showed a considerable current increment after glucose addition, which was attributed to the ability of the NCaPAni NW surface to adsorb a large number of glucose molecules. While the detection was performed in a PBS buffer at pH 7.0, the working range was limited to 10–120 μM. Table 2 displays other examples of non-enzymatic PBGS with PAni as sole transducer.

**Figure 11:**
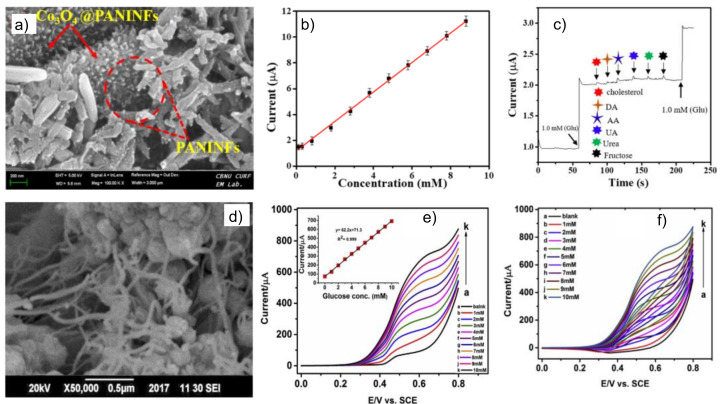
Non-enzymatic Co_3_O_4_@PaniNFs sensor: (**a**) FE-SEM view of composite, (**b**) calibration curve for glucose detection, and (**c**) selectivity of the device; PAni@CuNi sensor: (**d**) SEM image of the nanocomposite, glucose detection in 0.1 NaOH using LSV (**e**) and CV (**f**); The figure was adapted from references Bilal et al. [103] and Yassin et al. [104].

For improving the analytical features of detection, PAni combined with metallic nanoparticles (MNPs) has been applied for detection, Table 3. An essential function of MNPs is to increase the electronic transfer capacity of the PAni towards the electrode because this interaction enhances both the intrinsic conductivity and the surface area of the PAni. The electrocatalytic activity of MNPs is another elementary function in the design of glucose sensors. Its use allows solving some of the problems implied by enzymes, such as sensitivity to pH, temperature, the element of detection, and others. In this sense, a hybrid material, PAni@CuNi, was proposed by Bilal et al. [103] and obtained by mixing in the solution PAni synthesized by inverse emulsion polymerization and CuNi NPs synthesized by polyol method (Figure 11). The PAni provides a high surface area for NP growth, providing high catalytic activity towards glucose oxidation due to Ni oxidation states. The sensor exhibited a linear range from 0.1 to 5.6 mM, a sensitivity of 1030 µA mM^−1^ cm^−2^, and LOD of 0.2 µM. While the device was evaluated with blood serum samples, additional conditioning with an alkaline medium was necessary for detection. In a different approach, Yassin et al. [104] selected Co_3_O_4_, a semiconductor with electrocatalytic behavior and chemical stability, for a novel non-enzymatic sensor. Co_3_O_4_ showed a nanosheet morphology, subsequently incorporated in PAniNFs. The Co_3_O_4_@PAniNFs were cast onto the GCE, showing excellent electrical conductivity and a large active area. The addition of PAni allows a fast electron transfer and reversible redox process. An essential feature of this device is that glucose detection was performed in 0.1 M PBS (pH 7.4) as an electrolyte. The authors proposed that the registered anodic current due to glucose oxidation could be related to synergistic effects between PAni states and Co3O4 nanosheets. Additionally, the Co_3_O_4_@PAniNFs sensor achieved a linear detection range from 0.1 to 8 mM, operational stability after 27 days, and the ability of detection in blood serum samples.

**Figure 12:**
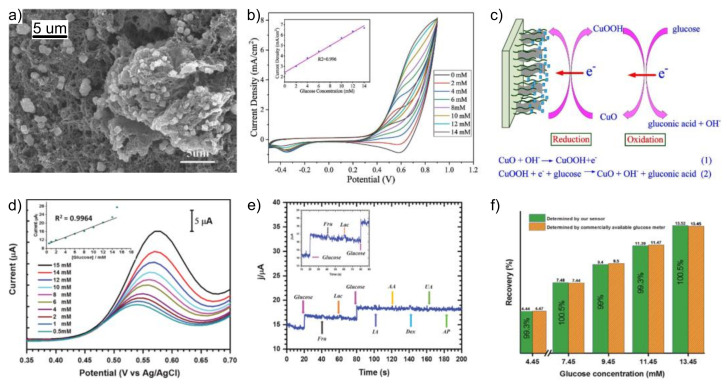
(**a**) SEM view of PAni/rGO/CuO NP sensor, (**b**) CV detection of glucose, (**c**) proposed sensing mechanism, (**d**) glucose detection with PANI(COOH)-PEI-Fc/Cu NP sensor using DPV and the obtained calibration curve, (**e**) selectivity of the device against common interferences, (**f**) performance against the commercial device. The figures were adapted with permissions from references Fang et al. [105] and Gopalan et al. [106].

The development of 3D structures increases the surface area and improves the electronic transmission efficiency of the electrode, Table 4. For example, Fang et al. obtained a 3D structure of PAni and rGO (Figure 12) [105]. PAni and GO were co-deposited by ECP on the electrode; then GO was electrochemically reduced to rGO, and the addition of electrodeposited CuO NPs completed the composite. Because the PAni conductivity is strongly influenced by pH, the alkaline electrolyte solution significantly impacts the performance of the composite, as observed by the authors in a composite without rGO. Thus, the combination of PAni with carbon nanomaterials improves the electrocatalytic activity of the proposed sensor, enhances the analytical parameters (linear range 0 to 13 mM), and promotes a fast response (<3 s). In a different approach, a multicomponent nanobead (MCNB) was developed using a copolymer of poly(aniline-*co*-anthranilic acid)-grafted-graphene (G-PANI(COOH), branched polyethyleneimine (b-PEI), and ferrocene carboxyaldehyde (Fc-CHO). All the components were integrated into a network structure further decorated with CuNPs. The G-PANI(COOH)-PEI-Fc/Cu NP assembly was applied for oxidation of glucose in 0.1 M PBS, allowing detection at lower potential (+0.37 V). The performance was attributed to the synergistic effects of MCNB assembly, which allowed large amounts of glucose absorption among enhanced characteristics. Glucose detection was performed in DPV, achieving a linear range from 0.5 to 14 mM [106].

**Table 2:** Non-enzymatic PAni-based glucose biosensors.

Material Electrode	Polymerization	Technique	Solution-pH	Linear Range	Sensitivity	LOD	Ref.
CPAniNS ^1^	COP	AMP	0.1 M H_2_SO_4_	1–1000 µM	2003.5 µA·mM^−1^·cm^−2^	0.043 µM	[107]
PAni/BioHAP/AgHg ^2^	COP	AMP	0.1 M PBS-7.4	1.0–10.0 mM	-	1.0 mM	[108]
NCa-PAni NWs ^3^	ECP	LSV ^4^	0.1 M PBS-7	10–120 µM	156.4 µA·mM^−1^·cm^−2^	0.657 µM	[102]

^1^ Conductive Polyaniline Nanosheets; ^2^ PAni/biomineralized hydroxyapatite/silver amalgam; ^3^ nanocages-augmented PANI nanowires; ^4^ LSV Linear Sweep Voltammetry.

**Table 3:** Non-enzymatic PAni/nanomaterials based glucose biosensors.

Material Electrode	Polymerization	Technique	Electrolyte	Linear Range	Sensitivity	LOD	Sample	Ref.
NiNPs/PAni	ECP	AMP	0.1 M NaOH	0.02–1 mM	278.8 µA·mM^−1^·cm^−2^	1 µM	-	[109]
CuNPs-Halloysite NT/PAni	COP	AMP	0.1 M NaOH	0.01–0.1; 0.1–0.5 mM	434.0; 57.7 µA·mM^−1^·cm^−2^	0.27 μM	-	[110]
PAni/Cd stannate/CHIT	COP	AMP	0.1 M NaOH	0.0005–0.01 M 0.01–0.5 M	5.23 µA·mM^−1^·cm^−2^; 1.32 µA·mM^−1^·cm^−2^	0.03 mM and 0.5 mM	-	[111]
NiFeNPs/PAni	COP	AMP	0.1 M NaOH	0.02–1 mM	1050 µA·mM^−1^·cm^−2^	0.5 μM	-	[112]
Polyacrilonitrile/ PAni/CuO	-	AMP	0.1 M NaOH	100 μM–3 mM	360 µA·mM^−1^·cm^−2^	1.2 μM	-	[113]
AuNPs-TiO_2_/PAni	ECP	AMP	0.1 M NaOH	0.01–10 mM	379.8 µA·mM^−1^·cm^−2^	0.15 μM	-	[114]
NiO/PAni	COP	AMP	0.1 M NaOH	0–100 μM	606.13 µA·mM^−1^·cm^−2^	0.19 µM	serum	[115]
NiONPs@PAniNS ^1^	COP	AMP	0.1 M NaOH	1–3000 μM	5625 µA·mM^−1^·cm^−2^	0.06 μM	serum	[116]
PdNW/3D-PAni	COP	AMP	0.1 M NaOH	5–9800 μM	146.6 µA·mM^−1^·cm^−2^	0.7 μM	serum	[117]
AuNPs-TiO_2_/PAni	ECP	AMP	0.1 M NaOH	0.01–8 mM	313.6 µA·mM^−1^·cm^−2^	0.15 μM	-	[118]
PAni/Ag nanoleaf	ECP	CV/AMP	0.1 M PBS	1–8 mM	4.3124 µA·mM^−1^·cm^−2^	0.06 μM	-	[119]
Cu–PAni	ECP	AMP	0.1 M NaOH	20 µM–1 mM	4140 µA·mM^−1^·cm^−2^	5 µM	-	[120]
Ni-SnO_x_/PAni/ CuO- cotton	COP	AMP	0.1 M NaOH	0.001–1; 1–10 mM	1625; 1325 µA·mM^−1^·cm^−2^	130 nM	-	[121]
Co_3_O_4_@PAniNFs	COP	AMP	0.1 M PBS	0.1–8 mM	14.25 µA·mM^−1^·cm^−2^	0.06 mM	serum	[104]
PAni/NiF ^2^	COP	AMP	0.10 M LiOH	1.0 μM–20 mM	237 µA·mM^−1^·cm^−2^	-	-	[122]
Hollow CuO/PAni NHFs ^3^	COP	AMP	0.1 M NaOH	0.001–19.899	-	0.45 µM	-	[123]
PAni@CuNi	inverse emulsion polymerization	AMP	0.1 M NaOH	0.1 to 5.6 mM	1030 µA·mM^−1^·cm^−2^	0.2 µM	serum	[103]
CuO/PAniNFs	COP	AMP	0.1 M NaOH	0.25 μM–0.28 mM; 0.28–4.6 mM	280; 1359 µA·mM^−1^·cm^−2^	0.24 μM	-	[124]
NiO/CuO/PAni	ECP	AMP	0.1 M NaOH	20–2500 µM	-	2 μM	serum	[125]
NiCo_2_O_4_@PAni	COP	AMP	0.1 M NaOH	0.015–4.7350 mM	4.55 mA·mM^−1^·cm^−2^	0.3833 μM	-	[126]
PAni/AuNPs	-	EIS	5 mM K_3_[Fe(CN)_6_]/0.1 M PBS	0.3–10 mM	-	0.1 mM	-	[127]

^1^ PAni nanosheets; ^2^ Nickel foam; ^3^ PAni nanohybrid fibers.

**Table 4:** Non-enzymatic PAni/Carbon-based materials/nanomaterials glucose biosensors.

Material Electrode	Polymerization	Technique	Electrolyte	Linear Range	Sensitivity	LOD	Sample	Ref.
ZnCo_2_O_4_/g-C_3_N_4_/Pani ^1^	COP	AMP	-	0.1–3 mM	15.64 mA·mM^−1^·cm^−2^	4 μM	-	[128]
PLA/GR/PAni/Cu	ECP	AMP	0.1 M KOH	1–7 mmol·L^−1^	-	49.3 mmol·L^−1^	-	[129]
CuNWs/PAni/rGO	-	-	-	0–4 mM	843.06 µA·mM^−1^·cm^−2^	1.6 mM	-	[130]
Cu^+2^/PAni/rGO ink	COP	AMP	0.1 M NaOH	2.8–22.2 µM; 0–4 mM	4168.37; 525.4 µA·mM^−1^·cm^−2^	4.93 µM	serum	[131]
PAniNS@rGO	COP	AMP	0.1 M NaOH	1–10 mM	3448.27 mA·mM^−1^·cm^−2^	30 nM	-	[107]
TiO_2_–rGO–PAni	COP	LSV	0.1 M NaOH	10–180 μM	-	7.46 μM	-	[132]
PAni/rGO/CuO	ECP	AMP	0.1 M NaOH	0–13 mM	1252 µA·mM^−1^·cm^−2^	1.5 μM	blood serum	[105]
PAni/ZnO/MWCNT	ECP	LSV	10 mM NaOH	0.1–1; 1–6 mM	7.8307; 1.6731 µA·mM^−1^·cm^−2^	0.1 μM	-	[133]
fGO/Fe_3_O_4_/PAni ^2^	COP	CV	PBS	0.05 μM–5 mM	-	0.01 μM	serum	[134]
NiCo2S4/rGO@PAni	COP	AMP	0.1 M NaOH	1–7000 μM	4.924 µA·µM^−1^·cm^−2^	0.14 μM	serum	[135]
NiO/Au/PAni/rGO	ECP	AMP	0.1 M NaOH	0.09–6 mM	-	0.23 μM	serum	[136]
AuNPs/PAni	ECP	CV	0.5 M KOH	10.26 μM–10.0 mM	150 μA·cm^−2^·mM ^−1^	3.08 μM	serum	[137]
PAni/CeO_2_ nanorods	-	AMP	0.1 M KCl	Up to 0.6 mM	25.79 µA·mM^−1^·cm^−2^	0.56 μM	-	[138]
CuNPs/PAni/graphene	COP	AMP	0.1 M NaOH	0.001–3.7 mM	150 mA·cm^−2^·M^−1^	0.27 μM	mice serum	[139]
G-PAni(COOH)-PEI-Fc/Cu-MCNB	COP	DPV	0.01 M PBS	0.50–14 mM	14.3 µA·mM^−1^·cm^−2^	0.16 mM	serum	[106]
NiONPs/PAniNW/GO	ECP	AMP	0.1 M NaOH	2 μM–5.560 mM	376.22 µA·mM^−1^·cm^−2^	0.5 μM	bovine serum	[140]

^1^ Zinc cobaltite/graphitic carbon nitride/PAni; ^2^ carboxy functionalized graphene oxide (fGO).

## 6. Conclusions and Future Directions

Glucose monitoring is an essential tool for the daily care of DM patients, and thus the development of accurate devices for detection is of high interest. PAni is a versatile CP with favorable characteristics for biomedical applications. So far, the majority of reviewed literature related to PAni applications in DM is focused on glucose sensing, using either enzymatic or non-enzymatic mechanisms. This is attributable to the intrinsic characteristics of PAni such as conductivity, biocompatibility, and tunable redox behavior, which allow fast, sensitive, and stable sensing devices. However, some significant challenges need to be addressed to propel PAni-based devices into a reality in DM care. Firstly, most of the sensors have been proposed from a material science perspective, rather than having a specific design to fit the parameters of biological samples. For example, blood glucose concentration in healthy individuals has been set in 5.6 mM, but for self-monitoring of diabetic patients, values up to 7 mM can be expected. While most of the reported PBGS presented an adequate LOD, the majority needed to increase their working range to fit the needs of blood glucose analysis. Specifically for non-enzymatic sensors, a key challenge is the dependence of alkaline electrolytes for glucose detection, which adds additional pre-processing steps and significantly impacts PAni conductivity. In this sense, non-enzymatic PBGS for detection in PBS similar to physiological conditions have been presented and need to be further improved. A growing and desirable field for DM is the continuous monitoring of glucose. PBGS have been developed for this purpose, achieving promising results for in vivo experiments. However, additional work for improving the biocompatibility of the entire electrochemical device needs to be addressed. A subject for further research is the trend of non-invasive, wearable devices capable of quantifying glucose in different body fluids such as urine, sweat, tears, and saliva. These alternative biological samples pose additional challenges such as low glucose concentration, a more comprehensive range of pH conditions, limited sample collection, and additional interferences. Few works related to the thematic have been presented for PBGS, but given the advances in manufacturing techniques, the polymer could be incorporated in novel wearable devices for on-body glucose detection. Besides glucose monitoring, the ability of PAni for switching between doped and undoped forms for acetone detection, one of the three ketone bodies related to diabetic ketoacidosis, has been explored. This property could be exploited for miniaturized sensors for both analytes with PAni as base material. Overall, this review has presented an overview of the current status of PBGS and the future directions for propelling this technology into DM care devices.

## Data Availability

Not applicable.

## References

[1] American Diabetes Association (2022). Introduction: Standards of Medical Care in Diabetes-2020. Diabetes Care.

[2] American Diabetes Association (2020). Classification and Diagnosis of Diabetes: Standards of Medical Care in Diabetes-2020. Diabetes Care.

[3] International Diabetes Federation IDF Diabetes Atlas, 10th ed.; Brussels, Belgium, 2021. https://diabetesatlas.org/idfawp/resource-files/2021/07/IDF_Atlas_10th_Edition_2021.pdf.

[4] Rubinstein I. (1984). Voltammetric PH Measurements with Surface-Modified Electrodes and a Voltammetric Internal Reference. Anal. Chem..

[5] Shinohara H., Chiba T., Aizawa M. (1988). Enzyme Microsensor for Glucose with an Electrochemically Synthesized Enzyme-Polyaniline Film. Sens. Actuators.

[6] Clark L.C., Lyons C. (1962). Electrode Systems for Continuous Monitoring in Cardiovascular Surgery. Ann. N. Y. Acad. Sci..

[7] Taguchi M., Ptitsyn A., McLamore E.S., Claussen J.C. (2014). Nanomaterial-Mediated Biosensors for Monitoring Glucose. J. Diabetes Sci. Technol..

[8] Zhu Z., Garcia-Gancedo L., Flewitt A.J., Xie H., Moussy F., Milne W.I. (2012). A Critical Review of Glucose Biosensors Based on Carbon Nanomaterials: Carbon Nanotubes and Graphene. Sensors.

[9] Choi Y.B., Kim H.S., Jeon W.Y., Lee B.H., Shin U.S., Kim H.H. (2019). The Electrochemical Glucose Sensing Based on the Chitosan-Carbon Nanotube Hybrid. Biochem. Eng. J..

[10] Dong Q., Ryu H., Lei Y. (2021). Metal Oxide Based Non-Enzymatic Electrochemical Sensors for Glucose Detection. Electrochim. Acta.

[11] Lakard B. (2020). Electrochemical Biosensors Based on Conducting Polymers: A Review. Appl. Sci..

[12] Nambiar S., Yeow J.T.W. (2011). Conductive Polymer-Based Sensors for Biomedical Applications. Biosens. Bioelectron..

[13] Chen C., Ran R., Yang Z., Lv R., Shen W., Kang F., Huang Z.H. (2018). An Efficient Flexible Electrochemical Glucose Sensor Based on Carbon Nanotubes/Carbonized Silk Fabrics Decorated with Pt Microspheres. Sens. Actuators B Chem..

[14] Cass A.E.G., Davis G., Francis G.D., Hill H.A. (1984). Determination of Glucose. Anal. Chem..

[15] Tsujimura S., Kojima S., Kano K., Ikeda T., Sato M., Sanada H., Omura H. (2006). Novel FAD-Dependent Glucose Dehydrogenase for a Dioxygen-Insensitive Glucose Biosensor. Biosci. Biotechnol. Biochem..

[16] Loughran M.G., Hall J.M., Turner A.P.F. (1996). Development of a Pyrroloquinoline Quinone (PQQ) Mediated Glucose Oxidase Enzyme Electrode for Detection of Glucose in Fruit Juice. Electroanalysis.

[17] Kenausis G., Taylor C., Katakis I., Heller A. (1996). “Wiring” of Glucose Oxidase and Lactate Oxidase within a Hydrogel Made with Poly(Vinyl Pyridine) Complexed with [Os(4,4′-Dimethoxy-2,2′-Bipyridine)2Cl]+/2+. J. Chem. Soc.—Faraday Trans..

[18] Nakabayashi Y., Omayu A., Yagi S., Nakamura K., Motonaka J. (2001). Evaluation of Osmium(II) Complexes as Electron Transfer Mediators Accessible for Amperometric Glucose Sensors. Anal. Sci..

[19] Jayakumar K., Bennett R., Leech D. (2021). Electrochemical Glucose Biosensor Based on an Osmium Redox Polymer and Glucose Oxidase Grafted to Carbon Nanotubes: A Design-of-Experiments Optimisation of Current Density and Stability. Electrochim. Acta.

[20] Arslan F., Beskan U. (2014). An Amperometric Biosensor for Glucose Detection from Glucose Oxidase Immobilized in Polyaniline-Polyvinylsulfonate-Potassium Ferricyanide Film. Artif. Cells Nanomed. Biotechnol..

[21] Kawaguri M., Yoshioka T., Nankai S. (1990). Disposable Glucose Sensor Employing Potassium Ferricyanide as a Mediator. Electrochem. Soc. Jpn..

[22] Heller A., Feldman B. (2008). Electrochemical Glucose Sensors and Their Applications in Diabetes Management. Chem. Rev..

[23] Heller A., Feldman B. (2010). Electrochemistry in Diabetes Management. Acc. Chem. Res..

[24] Bao S.J., Li C.M., Zang J.F., Cui X.Q., Qiao Y., Guo J. (2008). New Nanostructured TiO_2_ for Direct Electrochemistry and Glucose Sensor Applications. Adv. Funct. Mater..

[25] Guo C.X., Li C.M. (2010). Direct Electron Transfer of Glucose Oxidase and Biosensing of Glucose on Hollow Sphere-Nanostructured Conducting Polymer/Metal Oxide Composite. Phys. Chem. Chem. Phys..

[26] Diouf A., Bouchikhi B., el Bari N. (2019). A Nonenzymatic Electrochemical Glucose Sensor Based on Molecularly Imprinted Polymer and Its Application in Measuring Saliva Glucose. Mater. Sci. Eng. C.

[27] Caldara M., Lowdon J.W., Rogosic R., Arreguin-Campos R., Jimenez-Monroy K.L., Heidt B., Tschulik K., Cleij T.J., Diliën H., Eersels K. (2021). Thermal Detection of Glucose in Urine Using a Molecularly Imprinted Polymer as a Recognition Element. ACS Sens..

[28] Pletcher D. (1984). Electrocatalysis: Present and Future. J. Appl. Electrochem.

[29] Adzic R.R., Hsiao M.W., Yeager E.B. (1989). Electrochemical Oxidation of Glucose on Single Crystal Gold Surfaces. J. Electroanal. Chem..

[30] Burke L.D. (1994). Premonolayer Oxidation and Its Role in Electrocatalysis. Electrochim. Acta.

[31] Malhotra B.D., Chaubey A., Singh S.P. (2006). Prospects of Conducting Polymers in Biosensors. Anal. Chim. Acta.

[32] Gerard M., Chaubey A., Malhotra B.D. (2002). Application of Conducting Polymers to Biosensors. Biosens. Bioelectron..

[33] Zhao Y., Cao L., Li L., Cheng W., Xu L., Ping X., Pan L., Shi Y. (2016). Conducting Polymers and Their Applications in Diabetes Management. Sensors.

[34] Chiang J.C., MacDiarmid A.G. (1986). “Polyaniline”: Protonic Acid Doping of the Emeraldine Form to the Metallic Regime. Synth. Met..

[35] Huang W.S., Humphrey B.D., MacDiarmid A.G. (1986). Polyaniline, a Novel Conducting Polymer. Morphology and Chemistry of its Oxidation and Reduction in Aqueous Electrolytes. J. Chem. Soc. Faraday Trans. 1.

[36] Stafström S., Brédas J.L., Epstein A.J., Woo H.S., Tanner D.B., Huang W.S., MacDiarmid A.G. (1987). Polaron Lattice in Highly Conducting Polyaniline: Theoretical and Optical Studies. Phys. Rev. Lett..

[37] Kang E.T., Neoh K.G., Tan K.L. (1998). Polyaniline: A Polymer with Many Intrinsic Redox States. Prog. Polym. Sci..

[38] Molapo K.M., Ndangili P.M., Ajayi R.F., Mbambisa G., Mailu S.M., Njomo N., Masikini M., Baker P., Iwuoha E.I. (2012). Electronics of Conjugated Polymers (I): Polyaniline. Int. J. Electrochem. Sci..

[39] Kirova N. (2008). Understanding Excitons in Optically Active Polymers. Polym. Int..

[40] Catedral M.D., Tapia A.K.G., Sarmago R.V., Tamayo J.P., del Rosario E.J. (2004). Effect of Dopant Ions on the Electrical Conductivity and Microstructure of Polyaniline (Emeraldine Salt). Sci. Diliman.

[41] Canales M., Torras J., Fabregat G., Meneguzzi A., Alemán C. (2014). Polyaniline Emeraldine Salt in the Amorphous Solid State: Polaron versus Bipolaron. J. Phys. Chem. B.

[42] Monkman A.P., Bloor D., Stevens G.C., Stevens J.C.H. (1987). Electronic Energy Levels of Polyaniline. J. Phys. D Appl. Phys..

[43] Scotto J., Florit M.I., Posadas D. (2018). About the Species Formed during the Electrochemical Half Oxidation of Polyaniline: Polaron-Bipolaron Equilibrium. Electrochim. Acta.

[44] Macdiarmid A.G., Chiang J.C., Richter A.F., Epstein A.J. (1987). Polyaniline: A New Concept in Conducting Polymers. Synth. Met..

[45] Mu S., Xue H. (1996). Bioelectrochemical Characteristics of Glucose Oxidase Immobilized in a Polyaniline Film. Sens. Actuators B Chem..

[46] Yan W., Feng X., Chen X., Hou W., Zhu J.J. (2008). A Super Highly Sensitive Glucose Biosensor Based on Au Nanoparticles-AgCl @polyaniline Hybrid Material. Biosens. Bioelectron..

[47] Zhao M., Wu X., Cai C. (2009). Polyaniline Nanofibers: Synthesis, Characterization, and Application to Direct Electron Transfer of Glucose Oxidase. J. Phys. Chem. C.

[48] Zaks A., Klibanov A.M. (1988). Enzymatic Catalysis in Nonaqueous Solvents. J. Biol. Chem..

[49] Lukachova L.V., Karyakin A.A., Karyakina E.E., Gorton L. (1997). The Improvement of Polyaniline Glucose Biosensor Stability Using Enzyme Immobilization from Water-Organic Mixtures with a High Content of Organic Solvent. Sens. Actuators B Chem..

[50] Andrade J.D., Herron J., Lin J.N., Yen H., Kopecek J., Kopeckova P. (1988). On-Line Sensors for Coagulation Proteins: Concept and Progress Report. Biomaterials.

[51] Turner R.F.B., Harrison D.J., Rajotte R.V., Baltes H.P. (1990). A Biocompatible Enzyme Electrode for Continuous in Vivo Glucose Monitoring in Whole Blood. Sens. Actuators B. Chem..

[52] Rolfe P. (1990). In Vivo Chemical Sensors for Intensive-Care Monitoring. Med. Biol. Eng. Comput..

[53] Sun K.H., Liu Z., Liu C., Yu T., Shang T., Huang C., Zhou M., Liu C., Ran F., Li Y. (2016). Evaluation of in Vitro and in Vivo Biocompatibility of a Myo-Inositol Hexakisphosphate Gelated Polyaniline Hydrogel in a Rat Model. Sci. Rep..

[54] Humpolicek P., Kasparkova V., Saha P., Stejskal J. (2012). Biocompatibility of Polyaniline. Synth. Met..

[55] Kašpárková V., Humpoliček P., Stejskal J., Capáková Z., Bober P., Skopalová K., Lehocký M. (2019). Exploring the Critical Factors Limiting Polyaniline Biocompatibility. Polymers.

[56] Alim S., Kafi A.K.M., Rajan J., Yusoff M.M. (2019). Application of Polymerized Multiporous Nanofiber of SnO_2_ for Designing a Bienzyme Glucose Biosensor Based on HRP/GOx. Int. J. Biol. Macromol..

[57] Zeng X., Zhang Y., Du X., Li Y., Tang W. (2018). A Highly Sensitive Glucose Sensor Based on a Gold Nanoparticles/Polyaniline/Multi-Walled Carbon Nanotubes Composite Modified Glassy Carbon Electrode. New J. Chem..

[58] Maity D., Minitha C.R., Rajendra R.K. (2019). Glucose Oxidase Immobilized Amine Terminated Multiwall Carbon Nanotubes/Reduced Graphene Oxide/Polyaniline/Gold Nanoparticles Modified Screen-Printed Carbon Electrode for Highly Sensitive Amperometric Glucose Detection. Mater. Sci. Eng. C.

[59] Al-Sagur H., Komathi S., Khan M.A., Gurek A.G., Hassan A. (2017). A Novel Glucose Sensor Using Lutetium Phthalocyanine as Redox Mediator in Reduced Graphene Oxide Conducting Polymer Multifunctional Hydrogel. Biosens. Bioelectron..

[60] Zheng H., Liu M., Yan Z., Chen J. (2020). Highly Selective and Stable Glucose Biosensor Based on Incorporation of Platinum Nanoparticles into Polyaniline-Montmorillonite Hybrid Composites. Microchem. J..

[61] Yan Z., Zheng H., Chen J., Ye Y. (2019). The Micro Network of Polyacrylonitrile (PAN)-Polyaniline (Pani)-Graphene (GRA) Hybrid Nanocomposites for Effective Electrochemical Detection of Glucose and Improved Stability. Int. J. Electrochem. Sci..

[62] Yao Y., Wu S.G., Xu H.H., Wang L.W. (2015). High-Sensitive Glucose Biosensor Based on Ionic Liquid Doped Polyaniline/Prussian Blue Composite Film. Chin. J. Chem. Phys..

[63] Wu S., Su F., Dong X., Ma C., Pang L., Peng D., Wang M., He L., Zhang Z. (2017). Development of Glucose Biosensors Based on Plasma Polymerization-Assisted Nanocomposites of Polyaniline, Tin Oxide, and Three-Dimensional Reduced Graphene Oxide. Appl. Surf. Sci..

[64] Muthusankar E., Ragupathy D. (2019). Graphene/Poly(Aniline-Co-Diphenylamine) Nanohybrid for Ultrasensitive Electrochemical Glucose Sensor. Nano-Struct. Nano-Objects.

[65] Sassolas A., Blum L.J., Leca-Bouvier B.D. (2012). Immobilization Strategies to Develop Enzymatic Biosensors. Biotechnol. Adv..

[66] Lai J., Yi Y., Zhu P., Shen J., Wu K., Zhang L., Liu J. (2016). Polyaniline-Based Glucose Biosensor: A Review. J. Electroanal. Chem..

[67] Dhand C., Das M., Datta M., Malhotra B.D. (2011). Recent Advances in Polyaniline Based Biosensors. Biosens. Bioelectron..

[68] Zhang L., Zhou C., Luo J., Long Y., Wang C., Yu T., Xiao D. (2015). A Polyaniline Microtube Platform for Direct Electron Transfer of Glucose Oxidase and Biosensing Applications. J. Mater. Chem. B.

[69] Mousa H.M., Aggas J.R., Guiseppi-Elie A. (2019). Electropolymerization of Aniline and (N-Phenyl-o-Phenylenediamine) for Glucose Biosensor Application. Mater. Lett..

[70] Komathi S., Gopalan A.I., Muthuchamy N., Lee K.P. (2017). Polyaniline Nanoflowers Grafted onto Nanodiamonds via a Soft Template-Guided Secondary Nucleation Process for High-Performance Glucose Sensing. RSC Adv..

[71] Bicak T.C., Gicevičius M., Gokoglan T.C., Yilmaz G., Ramanavicius A., Toppare L., Yagci Y. (2017). Simultaneous and Sequential Synthesis of Polyaniline-g-Poly(Ethylene Glycol) by Combination of Oxidative Polymerization and CuAAC Click Chemistry: A Water-Soluble Instant Response Glucose Biosensor Material. Macromolecules.

[72] Feng X., Cheng H., Pan Y., Zheng H. (2015). Development of Glucose Biosensors Based on Nanostructured Graphene-Conducting Polyaniline Composite. Biosens. Bioelectron..

[73] Popov A., Aukstakojyte R., Gaidukevic J., Lisyte V., Kausaite-Minkstimiene A., Barkauskas J., Ramanaviciene A. (2021). Reduced Graphene Oxide and Polyaniline Nanofibers Nanocomposite for the Development of an Amperometric Glucose Biosensor. Sensors.

[74] Gautam V., Singh K.P., Yadav V.L. (2018). Polyaniline/Multiwall Carbon Nanotubes/Starch Nanocomposite Material and Hemoglobin Modified Carbon Paste Electrode for Hydrogen Peroxide and Glucose Biosensing. Int. J. Biol. Macromol..

[75] Miao Z., Wang P., Zhong A.M., Yang M., Xu Q., Hao S., Hu X. (2015). Development of a Glucose Biosensor Based on Electrodeposited Gold Nanoparticles-Polyvinylpyrrolidone-Polyaniline Nanocomposites. J. Electroanal. Chem..

[76] Chen D., Wang C., Chen W., Chen Y., Zhang J.X.J. (2015). PVDF-Nafion Nanomembranes Coated Microneedles for in Vivo Transcutaneous Implantable Glucose Sensing. Biosens. Bioelectron..

[77] Wu H., Shi C., Zhu Q., Li Y., Xu Z., Wei C., Chen D., Huang X. (2021). Capillary-Driven Blood Separation and in-Situ Electrochemical Detection Based on 3D Conductive Gradient Hollow Fiber Membrane. Biosens. Bioelectron..

[78] Hansen B., Hocevar M.A., Ferreira C.A. (2016). A Facile and Simple Polyaniline-Poly(Ethylene Oxide) Based Glucose Biosensor. Synth. Met..

[79] Homma T., Kondo M., Kuwahara T., Shimomura M. (2015). Polyaniline/Poly(Acrylic Acid) Composite Film: A Promising Material for Enzyme-Aided Electrochemical Sensors. Eur. Polym. J..

[80] Palsaniya S., Nemade H.B., Dasmahapatra A.K. (2019). Mixed Surfactant-Mediated Synthesis of Hierarchical PANI Nanorods for an Enzymatic Glucose Biosensor. ACS Appl. Polym. Mater..

[81] Zhu J., Liu X., Wang X., Huo X., Yan R. (2015). Preparation of Polyaniline-TiO_2_ Nanotube Composite for the Development of Electrochemical Biosensors. Sens. Actuators B Chem..

[82] Fang L., Liang B., Yang G., Hu Y., Zhu Q., Ye X. (2017). A Needle-Type Glucose Biosensor Based on PANI Nanofibers and PU/E-PU Membrane for Long-Term Invasive Continuous Monitoring. Biosens. Bioelectron..

[83] Cai Y., Liang B., Chen S., Zhu Q., Tu T., Wu K., Cao Q., Fang L., Liang X., Ye X. (2020). One-Step Modification of Nano-Polyaniline/Glucose Oxidase on Double-Side Printed Flexible Electrode for Continuous Glucose Monitoring: Characterization, Cytotoxicity Evaluation and in Vivo Experiment. Biosens. Bioelectron..

[84] Majumdar S., Mahanta D. (2020). Deposition of an Ultra-Thin Polyaniline Coating on a TiO_2_ surface by Vapor Phase Polymerization for Electrochemical Glucose Sensing and Photocatalytic Degradation. RSC Adv..

[85] Ma T.M., Zeng H., Zhao S.X., Huo W.S. (2019). Spectroscopic and Electrochemical Features of Glucose Oxidase Incorporation into Polyaniline-Cobaltous Oxalate Nano-Complex. J. Inorg. Organomet. Polym. Mater..

[86] Tang W., Li L., Zeng X. (2015). A Glucose Biosensor Based on the Synergistic Action of Nanometer-Sized TiO_2_ and Polyaniline. Talanta.

[87] Xia L., Xia J., Wang Z. (2015). Direct Electrochemical Deposition of Polyaniline Nanowire Array on Reduced Graphene Oxide Modified Graphite Electrode for Direct Electron Transfer Biocatalysis. RSC Adv..

[88] Mello H.J.N.P.D., Mulato M. (2020). Enzymatically Functionalized Polyaniline Thin Films Produced with One-Step Electrochemical Immobilization and Its Application in Glucose and Urea Potentiometric Biosensors. Biomed. Microdevices.

[89] Nogueira Pedroza Dias Mello H.J., Bueno P.R. (2020). Comparing Glucose and Urea Enzymatic Electrochemical and Optical Biosensors Based on Polyaniline Thin Films. Anal. Methods.

[90] Al-Sagur H., Shanmuga sundaram K., Kaya E.N., Durmuş M., Basova T.V., Hassan A. (2019). Amperometric Glucose Biosensing Performance of a Novel Graphene Nanoplatelets-Iron Phthalocyanine Incorporated Conducting Hydrogel. Biosens. Bioelectron..

[91] Al-Sagur H., Komathi S., Karakaş H., Atilla D., Gürek A.G., Basova T., Farmilo N., Hassan A.K. (2018). A Glucose Biosensor Based on Novel Lutetium Bis-Phthalocyanine Incorporated Silica-Polyaniline Conducting Nanobeads. Biosens. Bioelectron..

[92] German N., Ramanaviciene A., Ramanavicius A. (2021). Dispersed Conducting Polymer Nanocomposites with Glucose Oxidase and Gold Nanoparticles for the Design of Enzymatic Glucose Biosensors. Polymers.

[93] Neupane S., Bhusal S., Subedi V., Nakarmi K.B., Gupta D.K., Yadav R.J., Yadav A.P. (2021). Preparation of an Amperometric Glucose Biosensor on Polyaniline-Coated Graphite. J. Sens..

[94] Azimi S., Farahani A., Sereshti H. (2020). Plasma-Functionalized Highly Aligned CNT-Based Biosensor for Point of Care Determination of Glucose in Human Blood Plasma. Electroanalysis.

[95] Kuwahara T., Ogawa K., Sumita D., Kondo M., Shimomura M. (2018). Amperometric Glucose Sensing with Polyaniline/Poly(Acrylic Acid) Composite Film Bearing Glucose Oxidase and Catalase Based on Competitive Oxygen Consumption Reactions. J. Electroanal. Chem..

[96] Fathollahzadeh M., Hosseini M., Haghighi B., Kolahdouz M., Fathipour M. (2016). Fabrication of a Liquid-Gated Enzyme Field Effect Device for Sensitive Glucose Detection. Anal. Chim. Acta.

[97] Gangwar R.K., Dhumale V.A., Date K.S., Alegaonkar P., Sharma R.B., Datar S. (2016). Decoration of Gold Nanoparticles on Thin Multiwall Carbon Nanotubes and Their Use as a Glucose Sensor. Mater. Res. Express.

[98] Gong C., Chen J., Song Y., Sun M., Song Y., Guo Q., Wang L. (2016). A Glucose Biosensor Based on the Polymerization of Aniline Induced by a Bio-Interphase of Glucose Oxidase and Horseradish Peroxidase. Anal. Methods.

[99] Li R., Guo D., Ye J., Zhang M. (2015). Stabilization of Prussian Blue with Polyaniline and Carbon Nanotubes in Neutral Media for in Vivo Determination of Glucose in Rat Brains. Analyst.

[100] Zhu D., Nakamura H., Zhu H., Xu C., Matsuo M. (2015). Microfibers from Interpolymer Complexation of κ-Carrageenan and Oligomers of Polyaniline for Glucose Detection. Synth. Met..

[101] Hassan M.H., Vyas C., Grieve B., Bartolo P. (2021). Recent Advances in Enzymatic and Non-Enzymatic Electrochemical Glucose Sensing. Sensors.

[102] Ameen S., Akhtar M.S., Shin H.S. (2016). Nanocages-Augmented Aligned Polyaniline Nanowires as Unique Platform for Electrochemical Non-Enzymatic Glucose Biosensor. Appl. Catal. A Gen..

[103] Bilal S., Ullah W., Ali Shah A. (2018). ul H. Polyaniline@CuNi Nanocomposite: A Highly Selective, Stable and Efficient Electrode Material for Binder Free Non-Enzymatic Glucose Sensor. Electrochim. Acta.

[104] Yassin M.A., Shrestha B.K., Ahmad R., Shrestha S., Park C.H., Kim C.S. (2019). Exfoliated Nanosheets of Co3O4 Webbed with Polyaniline Nanofibers: A Novel Composite Electrode Material for Enzymeless Glucose Sensing Application. J. Ind. Eng. Chem..

[105] Fang L., Zhu Q., Cai Y., Liang B., Ye X. (2019). 3D Porous Structured Polyaniline/Reduced Graphene Oxide/Copper Oxide Decorated Electrode for High Performance Nonenzymatic Glucose Detection. J. Electroanal. Chem..

[106] Gopalan A.I., Muthuchamy N., Komathi S., Lee K.P. (2016). A Novel Multicomponent Redox Polymer Nanobead Based High Performance Non-Enzymatic Glucose Sensor. Biosens. Bioelectron..

[107] Kailasa S., Geeta B., Jayarambabu N., Kiran Kumar Reddy R., Sharma S., Venkateswara Rao K. (2019). Conductive Polyaniline Nanosheets (CPANINS) for a Non-Enzymatic Glucose Sensor. Mater. Lett..

[108] Jiang Y., Liu H., Qi X., Sun J., Li M., Wang J. (2018). Conductive Ag-Based Modification of Hydroxyapatite Microtubule Array and Its Application to Enzyme-Free Glucose Sensing. ChemistrySelect.

[109] Lakhdari D., Guittoum A., Benbrahim N., Belgherbi O., Berkani M., Seid L., Khtar S.A., Saeed M.A., Lakhdari N. (2021). Elaboration and Characterization of Ni (NPs)-PANI Hybrid Material by Electrodeposition for Non-Enzymatic Glucose Sensing. J. Electron. Mater..

[110] Luo Y., Liu W., Huang M., Zhang S., Zhao Y., Yang Q., Yan B., Gu Y., Chen S. (2021). Copper Nanoparticles Decorated Halloysite Nanotube/Polyaniline Composites for High Performance Non-Enzymatic Glucose Sensor. J. Electrochem. Soc..

[111] Bano S., Ganie A.S., Sultana S., Khan M.Z., Sabir S. (2021). The Non-Enzymatic Electrochemical Detection of Glucose and Ammonia Using Ternary Biopolymer Based-Nanocomposites. New J. Chem..

[112] Lakhdari D., Guittoum A., Benbrahim N., Belgherbi O., Berkani M., Vasseghian Y., Lakhdari N. (2021). A Novel Non-Enzymatic Glucose Sensor Based on NiFe(NPs)–Polyaniline Hybrid Materials. Food Chem. Toxicol..

[113] Jafari G., Gholipour-Kanani A., Rashidi A., Moazami H.R. Novel Non-Enzymatic Glucose Biosensor Based on Electrospun PAN/PANI/CuO Nano-Composites. J. Text. Inst..

[114] Chiu W.T., Chang T.F.M., Sone M., Tixier-Mita A., Toshiyoshi H. (2020). Roles of TiO_2_ in the Highly Robust Au Nanoparticles-TiO_2_ Modified Polyaniline Electrode towards Non-Enzymatic Sensing of Glucose. Talanta.

[115] Azharudeen A.M., Karthiga R., Rajarajan M., Suganthi A. (2020). Fabrication, Characterization of Polyaniline Intercalated NiO Nanocomposites and Application in the Development of Non-Enzymatic Glucose Biosensor. Arab. J. Chem..

[116] Kailasa S., Rani B.G., Bhargava Reddy M.S., Jayarambabu N., Munindra P., Sharma S., Venkateswara Rao K. (2020). NiO Nanoparticles -Decorated Conductive Polyaniline Nanosheets for Amperometric Glucose Biosensor. Mater. Chem. Phys..

[117] Li Z., Qian W., Guo H., Song X., Yan H., Jin R., Zheng J. (2020). Facile Preparation of Novel Pd Nanowire Networks on a Polyaniline Hydrogel for Sensitive Determination of Glucose. Anal. Bioanal. Chem..

[118] Chiu W.T., Chang T.F.M., Sone M., Tixier-Mita A., Toshiyoshi H. (2020). Electrocatalytic Activity Enhancement of Au NPs-TiO_2_ Electrode via a Facile Redistribution Process towards the Non-Enzymatic Glucose Sensors. Sens. Actuators B Chem..

[119] Mamlayya V.B., Maile N.C., Fulari V.J. (2020). A Study on Silver Nanoleaf-Decorated PANI Electrodes for Improved Electrochemical Performance. Polym. Bull..

[120] Belgherbi O., Chouder D., Lakhdari D., Dehchar C., Laidoudi S., Lamiri L., Hamam A., Seid L. (2020). Enzyme-Free Glucose Sensor Based on Star-Like Copper Particles-Polyaniline Composite Film. J. Inorg. Organomet. Polym. Mater..

[121] Sedighi A., Montazer M., Mazinani S. (2019). Synthesis of Wearable and Flexible NiP 0.1 -SnOx/PANI/CuO/Cotton towards a Non-Enzymatic Glucose Sensor. Biosens. Bioelectron..

[122] Liu K., Duan X., Yuan M., Xu Y., Gao T., Li Q., Zhang X., Huang M., Wang J. (2019). How to Fit a Response Current-Concentration Curve? A Semi-Empirical Investigation of Non-Enzymatic Glucose Sensor Based on PANI-Modified Nickel Foam. J. Electroanal. Chem..

[123] Liu T., Guo Y., Zhang Z., Miao Z., Zhang X., Su Z. (2019). Fabrication of Hollow CuO/PANI Hybrid Nanofibers for Non-Enzymatic Electrochemical Detection of H_2_O_2_ and Glucose. Sens. Actuators B Chem..

[124] Esmaeeli A., Ghaffarinejad A., Zahedi A., Vahidi O. (2018). Copper Oxide-Polyaniline Nanofiber Modified Fluorine Doped Tin Oxide (FTO) Electrode as Non-Enzymatic Glucose Sensor. Sens. Actuators B Chem..

[125] Ghanbari K., Babaei Z. (2016). Fabrication and Characterization of Non-Enzymatic Glucose Sensor Based on Ternary NiO/CuO/Polyaniline Nanocomposite. Anal. Biochem..

[126] Yu Z., Li H., Zhang X., Liu N., Tan W., Zhang X., Zhang L. (2016). Facile Synthesis of NiCo_2_O_4_@Polyaniline Core-Shell Nanocomposite for Sensitive Determination of Glucose. Biosens. Bioelectron..

[127] Ahammad A.J.S., al Mamun A., Akter T., Mamun M.A., Faraezi S., Monira F.Z. (2016). Enzyme-Free Impedimetric Glucose Sensor Based on Gold Nanoparticles/Polyaniline Composite Film. J. Solid State Electrochem..

[128] Kumar S., Fu Y.-P. (2021). PANI/g-C_3_N_4_ Composite over ZnCo_2_O_4_/Ni-Foam, a Bi-Functional Electrode as a Supercapacitor and Electrochemical Glucose Sensor. Sustain. Energy Fuels.

[129] Estadulho G.L.D., Alencar L.M., Guima K.E., Trindade M.A.G., Martins C.A. (2021). 3D-Printed Templates Converted into Graphite, Ruthenium, or Copper Are Used as Monolithic Sensors. ACS Appl. Electron. Mater..

[130] Anand V.K., Bhatt K., Kumar S., Archana B., Sharma S., Singh K., Gupta M., Goyal R., Virdi G.S. (2021). Sensitive and Enzyme-Free Glucose Sensor Based on Copper Nanowires/Polyaniline/Reduced Graphene Oxide Nanocomposite Ink. Int. J. Nanosci..

[131] Anand V.K., Bukke A., Bhatt K., Kumar S., Sharma S., Goyal R., Virdi G.S. (2020). Highly Sensitive and Reusable Cu+2/Polyaniline/Reduced Graphene Oxide Nanocomposite Ink-Based Non-Enzymatic Glucose Sensor. Appl. Phys. A Mater. Sci. Process..

[132] Du J., Tao Y., Xiong Z., Yu X., Xie A., Luo S., Li X., Yao C. (2019). Titanium Dioxide-Graphene-Polyaniline Hybrid for Nonenzymatic Detection of Glucose. Nano.

[133] Mohajeri S., Dolati A., Yazdanbakhsh K. (2019). Synthesis and Characterization of a Novel Non-Enzymatic Glucose Biosensor Based on Polyaniline/Zinc Oxide/Multi-Walled Carbon Nanotube Ternary Nanocomposite. J. Electrochem. Sci. Eng..

[134] Batool R., Akhtar M.A., Hayat A., Han D., Niu L., Ahmad M.A., Nawaz M.H. (2019). A Nanocomposite Prepared from Magnetite Nanoparticles, Polyaniline and Carboxy-Modified Graphene Oxide for Non-Enzymatic Sensing of Glucose. Microchim. Acta.

[135] Wang Y., Zhong J., Ding F., Zhao Q., Zhang Z., Liu X., Liu Y., Rao H., Zou P., Wang X. (2018). A Bifunctional NiCo2S4/Reduced Graphene Oxide@polyaniline Nanocomposite as a Highly-Efficient Electrode for Glucose and Rutin Detection. New J. Chem..

[136] Ghanbari K., Ahmadi F. (2017). NiO Hedgehog-like Nanostructures/Au/Polyaniline Nanofibers/Reduced Graphene Oxide Nanocomposite with Electrocatalytic Activity for Non-Enzymatic Detection of Glucose. Anal. Biochem..

[137] Xu M., Song Y., Ye Y., Gong C., Shen Y., Wang L., Wang L. (2017). A Novel Flexible Electrochemical Glucose Sensor Based on Gold Nanoparticles/Polyaniline Arrays/Carbon Cloth Electrode. Sens. Actuators B Chem..

[138] Zhang J., Guan P., Li Y., Li W., Guo Q. (2016). Polyaniline/Cerium Oxide Hybrid Modified Carbon Paste Electrode for Non-Enzymatic Glucose Detection. Bull. Korean Chem. Soc..

[139] Zheng W., Hu L., Lee L.Y.S., Wong K.Y. (2016). Copper Nanoparticles/Polyaniline/Graphene Composite as a Highly Sensitive Electrochemical Glucose Sensor. J. Electroanal. Chem..

[140] Zhuang X., Tian C., Luan F., Wu X., Chen L. (2016). One-Step Electrochemical Fabrication of a Nickel Oxide Nanoparticle/Polyaniline Nanowire/Graphene Oxide Hybrid on a Glassy Carbon Electrode for Use as a Non-Enzymatic Glucose Biosensor. RSC Adv..

